# Wastewater-based epidemiology for public health – benefits and trade-offs of different molecular methods for the generation of actionable data in a small-town context

**DOI:** 10.3389/fpubh.2026.1828355

**Published:** 2026-06-22

**Authors:** Ivana Kraiselburd, Ruzica Susenburger-Lange, Miriam Balzer, Simon Magin, Katharina Block, Leah Consten, Adrian Dörr, Ann-Kathrin Dörr, Jule Gosch, Shivohum Nishad, Sven Sachse, Alexander Thomas, Alexander Triebs, Josefa Welling, Alexander Wilhelm, Marek Widera, Ricarda Schmithausen, Folker Meyer

**Affiliations:** 1 Institute for AI in Medicine, University Hospital Essen, University of Duisburg-Essen, Essen, Germany; 2 Gesundheitsamt Mettmann, Mettmann, Germany; 3 Institute for Medical Virology, University Hospital, Goethe University Frankfurt, Frankfurt am Main, Germany

**Keywords:** antimicrobial resistance genes, infectious diseases, metagenomics, one health, public health

## Abstract

**Background:**

Wastewater-based epidemiology (WBE) is a promising complement to traditional surveillance systems, yet its practical utility and performance in real-world public health settings remain insufficiently characterized. This study aims to evaluate the feasibility and added value of WBE for monitoring infectious disease dynamics at the regional level, with a particular focus on jointly identifying, together with public health authorities, actionable and scalable methodological strategies based on cost, applicability, and the relevance and timeliness of the information generated.

**Methods:**

Composite influent wastewater samples were collected over 6 weeks from a treatment plant serving a defined district in western Germany. Samples were analyzed using quantitative PCR and both targeted and shotgun metagenomic sequencing. WBE findings were compared with routine case-based surveillance data from the corresponding catchment area.

**Results:**

All pathogens reported through routine public health surveillance during the study period were also detected in wastewater. In addition, WBE identified signals from clinically relevant pathogens not captured by case-based surveillance. Sequencing approaches provided further resolution on pathogen diversity and resistance profiles. The combined use of targeted and untargeted methods revealed differences in sensitivity and resolution, with complementary strengths across approaches, and enabled the definition of a practical, tiered approach to support actionable surveillance at the regional level.

**Conclusion:**

This study describes the operational integration of WBE into a regional public health workflow, providing timely, population-level data that complements routine surveillance and can reveal pathogen circulation not captured by reported cases. Building on the established advantages of WBE, our results highlight its practical value when jointly implemented with public health authorities, enabling context-specific, actionable insights that enhance situational awareness, guide targeted local responses and support earlier detection of emerging threats.

## 1 Introduction

As our knowledge rapidly expanded during the SARS-CoV-2 pandemic, fueled by unprecedented data availability, it became evident that zoonotic spillovers are not rare events but occur regularly (1). This finding, alongside other looming threats (2, 3), underscores the urgency of leveraging all available biomedical tools to minimize the future impact of infectious diseases.

Wastewater-based epidemiology (WBE) re-emerged as a valuable addition to individual public health surveillance during the COVID-19 pandemic (4). In Germany, for example, the national wastewater surveillance program AMELAG (5) was jointly developed by the Robert Koch Institute and the Federal Environment Agency for integrating data from wastewater treatment plants to support population-level epidemiological assessment. This program employed quantitative RT-qPCR to monitor and report the presence of SARS-CoV-2 in samples from up to 169 wastewater treatment plants, each serving approximately 185,000 individuals. AMELAG and other similar projects showed that continuous wastewater sampling provides an integral picture of the development of infection events: both the amount of SARS-CoV-2 virus circulating and the development of virus variants are reflected in untreated municipal wastewater (6), which complements mandatory clinical reporting systems.

Despite national-scale deployments, the consistent application of WBE in small-town contexts to generate actionable intelligence for local health authorities remains underexplored. Integrating WBE data with AI-based approaches can enable early warning systems for pathogen abundance changes (7, 8) supporting timely public health decision-making. Such systems, combined with clinical reporting obligations, offer strong predictive power.

PCR based approaches (9) and direct sequencing of environmental samples (10) are well suitable to characterize microbial, viral and fungal organisms across environmental and human-associated contexts. The low cost, quick turnaround nature of qPCR has demonstrated its utility in the COVID-19 pandemic (11), yet its potential to enrich local-level public health insights remains insufficiently studied. Untargeted strategies such as shotgun metagenomics enable the unbiased discovery of a variety of different organisms, including those not captured by predefined surveillance panels. In fact, in this study, we identified several microorganisms relevant to human health that, although notifiable, were not detected through routine surveillance, underscoring the added value of sequencing-based approaches for comprehensive monitoring.

In addition to viral pathogens, bacterial infections are a substantial threat to public health. With antibiotic resistance steadily increasing, bacterial infections are a significant burden on healthcare systems (12). Conventional culture-based susceptibility testing is inherently time-intensive, whereas clinical management of severe bacterial infections is o#en time-critical. At the same time, minimizing unnecessary broad-spectrum antibiotic use is essential to limit selective pressure and reduce the further emergence and spread of resistance. Wastewater surveillance enables the simultaneous monitoring of circulating bacterial species and associated resistance determinants, providing population-level insight into emerging resistance patterns. In outbreak situations, such information may support earlier optimization of empirical therapy.

In this study, we applied WBE to a small district in Germany. We employed qPCR for the targeted detection of antimicrobial resistance genes (ARGs) and of SARS-CoV-2 as an example of viral pathogens. In addition, we performed targeted and shotgun metagenomic sequencing for screening bacterial pathogens and their associated antibiotic resistance genes. Given the high dimensionality of metagenomic data, we prioritized bacteria included in the World Health Organization (WHO) 2024 priority pathogens list (13) to focus the analysis on organisms of established clinical relevance. To assess the practical utility and predictive value of WBE in this small-town setting, we compared wastewater findings with contemporaneous notifiable disease data in collaboration with regional public health authorities. The main aim was to assess the feasibility of implementing wastewater surveillance as a complementary tool for monitoring infection dynamics at the regional catchment level, and specifically, to identify actionable strategies for local public health decision-making by systematically comparing qPCR, targeted metagenomics and shotgun metagenomics in terms of applicability, information yield and complexity.

## 2 Materials and methods

### 2.1 Study design

This study combines targeted qPCR-based methods with targeted and untargeted metagenomics in a small district in north-western Germany outside of primary respiratory virus season in 2024. We applied methodology established in the context of the German AMELAG (5) effort, specifically the qPCR and RT-dPCR protocols designed during the WBEready research project. Details about the WBEready project can be found in the Supplementary information. We focused on a defined set of priority pathogens. SARS-CoV-2 virus was evaluated as clinically and epidemiologically relevant viral target. For bacterial pathogens and antimicrobial resistance genes, the analysis was centered on species included in the 2024 bacterial priority pathogens list of the WHO (13). In addition, untargeted metagenomic screening allowed the identification and reporting of other relevant pathogens detected beyond this predefined list.

Wastewater samples were collected weekly over a six-week period (20 August 2024 to 23 September 2024) from the influent of a municipal WWTP located in Monheim/Langenfeld, receiving discharges from the city of Langefeld, county of Mettman, Germany. Langenfeld has a population of 59,975 inhabitants (2024), with two hospitals (one for general medicine and one for psychiatry), eight residential care facilities for older adults, 17 schools, and 29 daycare centers. Rainwater and wastewater are fed separately into the sewer system. This gives the valuable opportunity of testing infectious agents in rainwater-free wastewater, avoiding potential dilution effects.

An automated mobile sampler (MAXX P6-L) was programmed to collect subsamples every 2 h for 24 h; samples were not normalized for flow. Subsamples were combined into composite samples and transported at 4 °C to the laboratory within 24 h.

### 2.2 Input from notifiable diseases in the Mettmann County

Under the Infection Protection Act, municipalities in Germany are mandated to report notifiable infectious diseases through a standardized surveillance framework. In this system, medical providers submit case notifications to local public health authorities as part of routine statutory surveillance. The data used in this study consisted of aggregated case counts without patient-identifying information. These data were obtained through a formal cooperation agreement with the Mettmann County Health Department. Weekly counts of reported infections, including multidrug-resistant organism (MRE) infections, COVID-19, influenza, respiratory syncytial virus (RSV), and measles, were provided. Cases reported are restricted to those arising within the catchment area of the sampled wastewater treatment plant. To address potential temporal misalignment between clinical case reporting and wastewater measurements, surveillance data from both the preceding and subsequent weeks relative to each sampling time point were incorporated into the analysis.

### 2.3 Laboratory sample preparation and nucleic acid extraction

Wastewater samples were incubated overnight at 4 °C to allow solid decantation. After decantation, two 100 μL aliquots of the supernatant were filtered through 0.45 μm electronegative filters (MF-Millipore) placed on stainless steel holders (Sartorius) using pressurized air. Filters were cut and placed into innuSPEED Lysis Tubes (IST Innuscreen) with 1 mL of DNA/RNA Shield (Zymo Research) and processed on a FastPrep-24 5G Instrument (MP Biomedicals). Lysis tubes were centrifuged at 10,000 g for 2 min. The supernatant was adjusted to 400 μL and used for automated nucleic acid extraction with the innuPREP AniPath DNA/RNA Kit on an InnuPure C16 device (Analytik Jena). The elution volume was set to 125 μL. Sterile MilliQ water served as a negative control.

### 2.4 RT-dPCR analysis

Digital RT-PCR was carried out for SARS-CoV-2 detection using the QIAcuity OneStep Advanced Probe Kit (Qiagen, Hilden, Germany) in combination with the QIAcuity Digital PCR System (Qiagen, Hilden, Germany), following previously described protocols (14) and using primer and probes specific for the genes N1 and N2 of SARS-CoV-2 (EP application no. EP25165787.0). Briefly, 15 μL of RNA were used per reaction, conducted in a total volume of 40 μL per reaction with two technical replicates on QIAcuity nanoplate 26 k 24-well plates (Qiagen, Hilden, Germany). Data analysis was performed using the QIAcuity Software Suite version 1.2.18 (Qiagen, Hilden, Germany). The digital RT-PCR output was expressed as copies per microliter of extracted RNA. These values were converted to genome copies per liter of wastewater by accounting for sample concentration and extraction factors applied prior to the RT-dPCR analysis.

### 2.5 qPCR analyses for antibiotic resistance genes

For the detection antibiotic resistance genes, qPCR reactions were prepared with Luna® Universal qPCR Master Mix (NEB M3003) in a total volume of 20 μL and run on a qTower3 Real-Time Thermal Cycler (Analytics Jena) using SYBR Green detection. Reactions were initiated with a 1-min activation at 95 °C, followed by 40 PCR cycles. Two cycling schemes were applied depending on the primer set: (1) 10 s denaturation at 95 °C and 30 s annealing/elongation at 60 °C; (2) 10 s denaturation at 95 °C, 15 s annealing at 55 °C, and 30 s elongation at 60 °C. qPCR efficiency was assessed using positive controls and serial dilutions. See Supplementary Table S1 for details on primers.

### 2.6 SARS-CoV-2 tiled amplicon sequencing

SARS-CoV-2 whole (meta-)genome sequencing was performed as previously described (15). After cDNA generation with LunaScript-RT-SuperMix (NEB), sequencing libraries were prepared using the EasySeq™ SARS-CoV-2 Whole Genome NGS Sequencing Kit (Nimagen) and sequenced on an Illumina MiSeq (V2 chemistry, 300 cycles). Raw FASTQ files were analyzed using the open-source UnCoVar pipeline (16). The pipeline conducted quality control, adapter and primer trimming, removal of low-quality reads and human contamination filtering. Reads were mapped to the Wuhan SARS-CoV-2 reference genome (NC_045512.2 or MN908947) and abundant lineages were evaluated with Freyja (17). The computational work for tiled amplicon analysis can be carried out on a single Laptop computer running either Linux or Windows with WSL.

### 2.7 Targeted 16S rDNA metagenomics analysis of bacteria

Targeted metagenomic analysis was performed by characterizing microbial communities using 16S rDNA sequencing. The preparation of sequencing libraries was performed following the 16S rDNA Metagenomic sequencing Library Preparation protocol (Illumina). Primers Bakt_341F and Bakt_805R targeting the V3-V4 region were used. These libraries were sequenced on an Illumina MiSeq (V2 chemistry, 500 cycles).

The resulting raw FASTQ files were processed using the QIIME2-based (18) open-source pipeline RiboSnake (19). After quality control and filtering, reads were clustered into Operational Taxonomic Units (OTUs) and taxonomically classified based using the SILVA database, version 138 (20). Each 16S rDNA dataset yielded between 130,000 and 170,000 reads. The computational work for the subsequent analysis can be carried out on a single Laptop computer running either Linux or Windows with WSL.

### 2.8 Shotgun metagenomics analysis of bacteria

Shotgun metagenomic sequencing libraries were generated using the Nextera XT DNA Library Prep kit (Illumina) and sequenced on an Illumina NextSeq 2000 (standard SBS P3 chemistry, 300 cycles).

Raw FASTQ files were processed using an automated Snakemake (21) pipeline performing quality control, adapter and primer trimming, removal of low-quality reads, and filtering of human contamination. Subsequently, it integrates MEGAHIT (22) for assembly and a combination of MetaBAT (23), MetaCoAG (24) and DASTool (22) for binning. The resulting Metagenome-Assembled Genomes (MAGs) were evaluated for quality with CheckM2 (25). Taxonomic classification on reads was performed using Kaiju (26) and on MAGs using GTDB-Tk (27). ARGs were identified using the CARD (28) database for contigs and MAGs. Each metagenome generated between 211 and 342 million reads. The computational work for shotgun metagenomics requires a Linux server with at least 128GB RAM, 2 Terabyte free disk space and 32 cores.

## 3 Results

### 3.1 Data from the existing surveillance system

**Figure 1:**
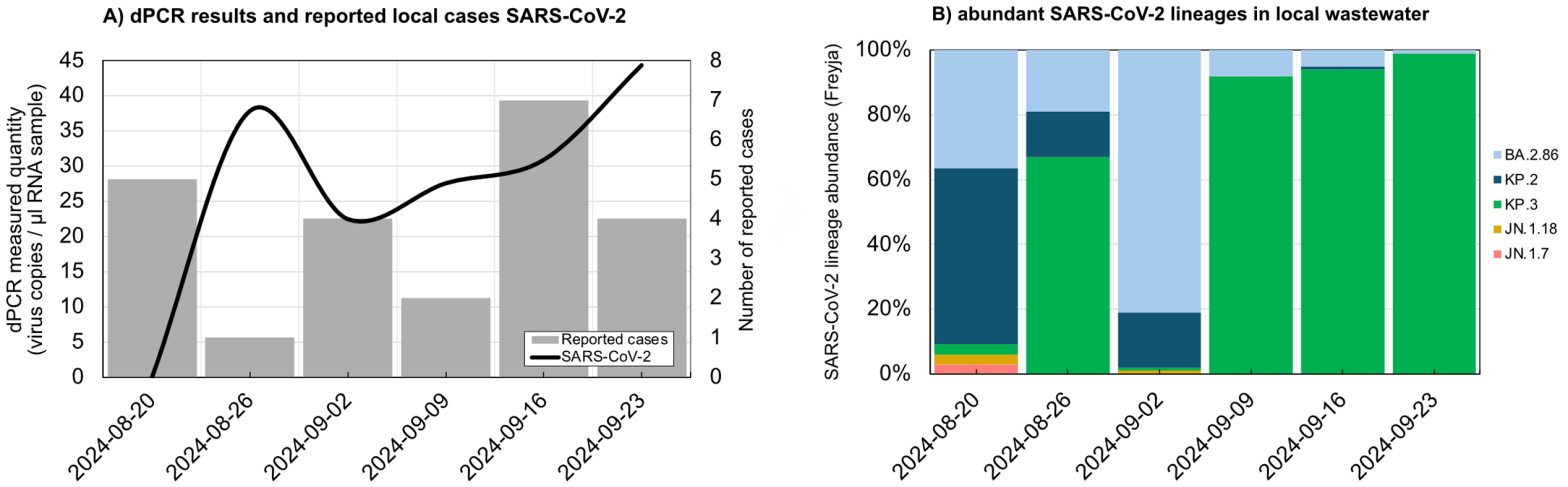
**(A)** Determination of SARS-CoV-2 RNA genome copy equivalents using digital RT-PCR compared to the number of locally reported patient cases per week during the sampling period of 6 weeks. Results are shown for quantification based on SARS-CoV-2 N1 gene. These values together with those calculated based on the N2 gene are presented in Supplementary Table S2. The corresponding viral copy numbers per liter of processed wastewater are also depicted in the Supplementary information. **(B)** SARS-CoV-2 lineages detected in wastewater. Except for the first and the third sampling points, the KP.3 variant is the dominant one. For the first sampling point it’s KP.2 and for the third it’s BA.2.86.

For the city of Langefeld, in Mettmann county, a total of 23 Covid-19 cases were reported during the period in question (Figure 1A). To include time delays between reporting and detection in the wastewater, the cases 1 week before and 1 week after the wastewater sampling period were also included in the first and last week. During the resulting 8-week period, a total of 30 coronavirus cases were reported. Besides reported SARS-CoV-2 cases, 2 MDRO (multidrug-resistant organisms) Enterobacterales patients were reported, one in the week of September 2nd and one in the week of September 9th. Reporting of Enterobacterales with Carbapenem resistance is mandatory (29). In the second case, the organism was identified as *Klebsiella pneumoniae* exhibiting carbapenem resistance. This corresponds to the WHO classification of carbapenem-resistant Enterobacterales as a high-risk pathogen (13). In the first case, no carbapenem resistance was reported and additional resistance profiles were not assessed. The organism was not further characterized.

### 3.2 SARS-CoV-2 evaluation by digital RT-PCR and tiled amplicon sequencing

Figure 1 shows the SARS-CoV-2 RNA genome copy equivalents in RNA extracted from wastewater samples as determined by digital RT-PCR compared to the reported cases (Figure 1A) and the relative abundance of the different variances measured by SARS-CoV-2 lineages (Figure 1B). SARS-CoV-2 was detected in wastewater at nearly all timepoints, with genome copy equivalents showing considerable variation, indicating that the amount of circulating virus also fluctuated. In addition to RT-dPCR-based quantification, viral genotypic characterization of the wastewater samples was performed through SARS-CoV-2 tiled amplicon sequencing as previously described in Schmiege *et al*. (15). and Thomas *et al*. (16) (Figure 1B). The national dominance of the KP.3 variant was also observed in samples from the local wastewater treatment plant, reflecting the epidemiological trend.

### 3.3 Antibiotics resistance genes from qPCR and shotgun metagenomics

**Figure 2:**
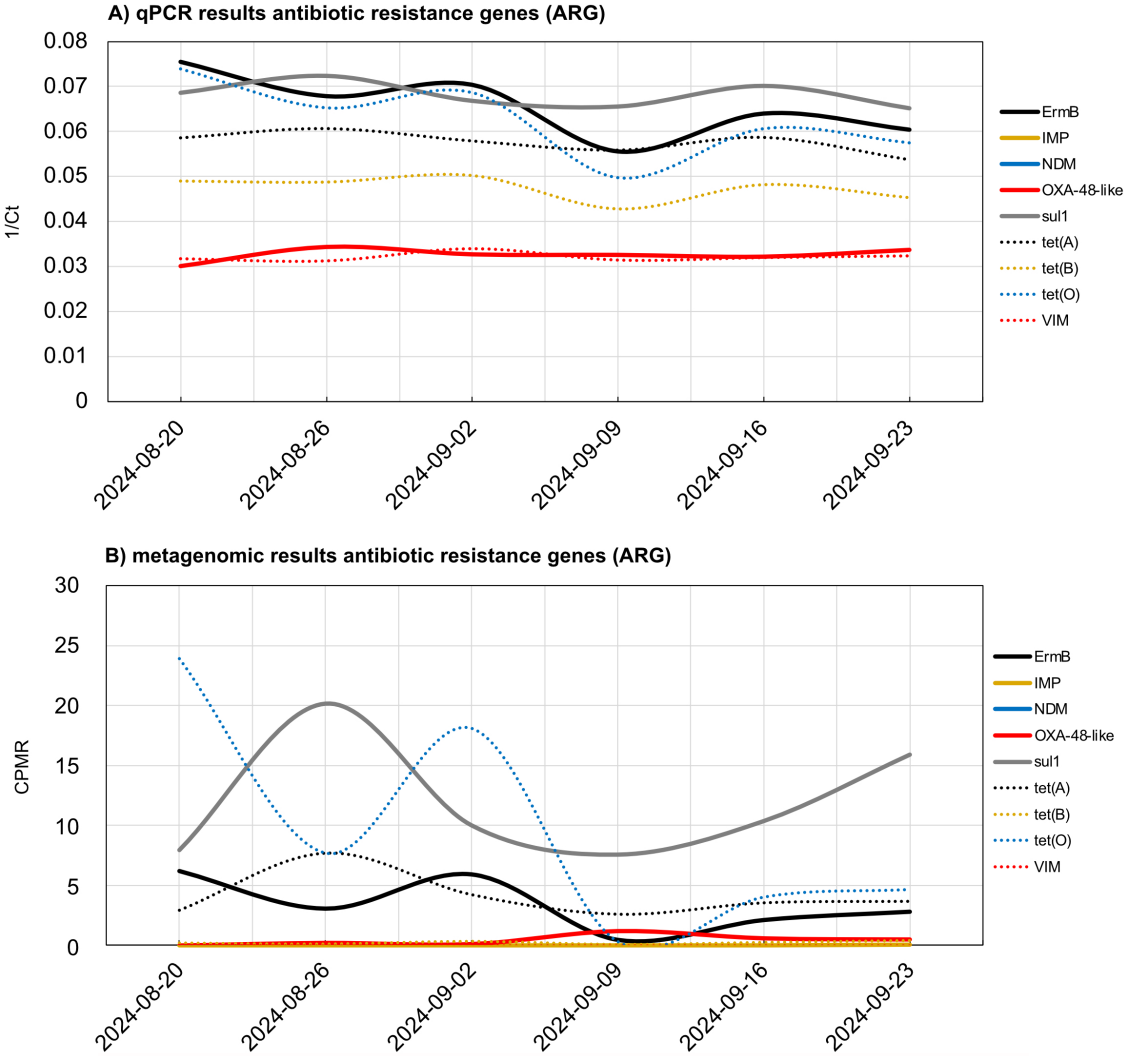
Comparison of ARG abundance determined by qPCR and shotgun metagenomics. **(A)** Reciprocal Ct values for the ARGs VIM, OXA-48-like, *sul1*, *ErmB*, *tetA*, *tetB*, *tetO*, NDM, and IMP measured during the sampling period. CT values are presented in Supplementary Table S2. **(B)** Relative abundance of the same ARGs in shotgun metagenomic data.

Using qPCR, we detected seven antibiotic resistance genes in wastewater: VIM, OXA-48-like, *sul1*, *ErmB*, *tetA*, *tetB*, and *tetO* (Figure 2A). The gene families VIM and OXA-48-like encode carbapenem resistance and are frequently associated with hospital outbreaks. *sul*1 confers resistance to sulfonamides, *ErmB* to macrolides, lincosamides, and streptogramin B, while *tetA* and *tetB* encode efflux pumps mediating tetracycline resistance. In addition, *tetO* encodes a ribosomal protection protein that also confers tetracycline resistance (Table 1). We further screened for IMP and NDM gene families, which are associated with carbapenem resistance, but these genes were not detected in any of the wastewater samples. qPCR Ct values are presented in Supplementary Table S2.

**Table 1:** Table summarizing the identified antibiotic resistance genes, the number of detected variants per gene, and their corresponding antibiotic drug classes, as defined by the Comprehensive Antibiotic Resistance Database [CARD, (28)].

Gene (family)	No. of variants	Inhibited drug class
ErmB	1	Streptogramin antibiotic Streptogramin B antibiotic Streptogramin A antibiotic Lincosamide antibiotic Macrolide antibiotic
IMP	102	Penicillin beta-lactam Cephalosporin Carbapenem
NDM	71	Penicillin beta-lactam Cephalosporin Carbapenem
OXA-48-like	67	Penicillin beta-lactam Carbapenem
*sul*1	1	Sulfonamide antibiotic
*tet*A	1	Tetracycline antibiotic
*tet*B	1	Tetracycline antibiotic
*tet*O	1	Tetracycline antibiotic
VIM	92	Penicillin beta-lactam Cephalosporin Carbapenem

In addition, contigs reconstructed from shotgun metagenomic sequencing were analyzed using the Resistance Gene Identifier (RGI) tool (28) to screen for the same set of genes (Figure 2B). Consistent with the qPCR results, NDM and IMP were detected in only one to two samples and at very low relative abundance. In contrast to the qPCR findings, VIM was not detected in the metagenomic data. All other targeted resistance genes were identified in nearly every sample, indicating their widespread distribution in the wastewater metagenome. The abundance estimates derived from metagenomics showed substantially greater variance compared to qPCR measurements. In particular, *sul*1 fluctuated between 8 and 20 copies per million reads (CPMR), and *tetO* ranged from 0 to nearly 25 CPMR. To relate detected genes to resistance phenotypes, Table 1 summarizes the number of variants identified per gene and the antibiotic classes they are predicted to confer resistance against. Detailed read counts and CPMR values are provided in Supplementary Table S3.

### 3.4 Detection of bacterial pathogens

#### 3.4.1 ESKAPE pathogens in targeted and untargeted metagenomics

We applied two methods for bacterial detection in this study: targeted 16S rDNA sequencing and shotgun metagenomics. In contrast to shotgun metagenomics, 16S rDNA analysis does not provide reliable taxonomic resolution beyond the genus level. To maintain the highest possible resolution of metagenomics, comparisons between the two methods were performed at different levels of taxonomic resolution.

**Figure 3:**
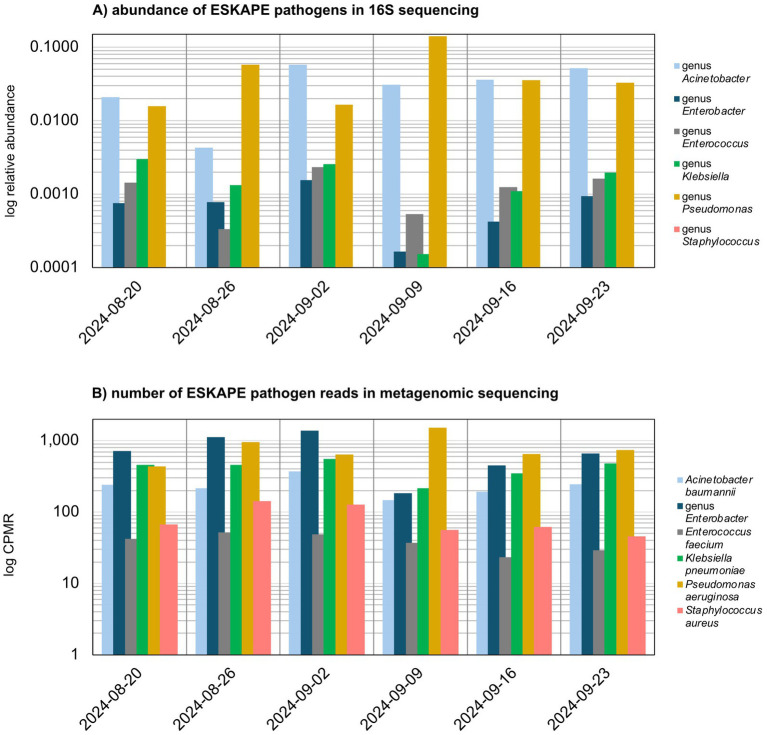
Comparison of the relative abundance of ESKAPE pathogen abundance with 16S rDNA targeted **(A)** and shotgun metagenomics **(B)**. A significant number of ESKAPE pathogen reads are present at all timepoints.

We observed different abundances for the various ESKAPE pathogens when using 16S rDNA gene-based approaches (Figure 3A) compared to shotgun metagenomics (Figure 3B). The 16S rDNA analysis does not show the genus *Staphylococcus*, while the analysis with shotgun metagenomics clearly indicates the presence of *Staphylococcus aureus*. On the other hand, 16S rDNA sequencing leads to an overrepresentation of the genus *Pseudomonas* and *Acinetobacter* compared to the shotgun metagenomics approach.

#### 3.4.2 WHO bacterial priority pathogens and their antibiotic resistances in metagenome assembled genomes

In total, 480 metagenome-assembled genomes (MAGs) were reconstructed from the shotgun metagenomic sequencing data. Of these, 38 were assigned to the order Enterobacterales. Additionally, 20 MAGs were taxonomically classified as members of species included in the WHO bacterial priority pathogen list. Resistance genes were detected in all these MAGs. It is likely that further priority organisms were present but could not be resolved at the species level.

MAGs corresponding to members of the WHO priority pathogen list were detected at nearly all sampling time points, except for week 4, when no suitable MAGs could be recovered. Most identified MAGs were assigned to the critical priority group, predominantly within the order Enterobacterales. Although detailed species-level classifications for this order are not presented in Table 2, several MAGs could be resolved to species level, including *Klebsiella pneumoniae* harboring carbapenem resistance. As the WHO list categorizes carbapenem-resistant Enterobacterales as critical priority pathogens, species-level assignments are provided in Supplementary Table S4 for a comprehensive view. The traditional case-based surveillance system identified a carbapenem resistant *K. pneumoniae* in the week of September 9th. While genome sequencing of the strain isolated form the patient would be needed for corroboration, or results suggest this bacterium could have been already detected in wastewater at the first sampling point, on August 20th.

**Table 2:** Number of metagenome-assembled genomes (MAGs) matching WHO bacterial priority pathogens on different sampling dates (+ potential matching MAGs).

WHO group	Taxonomy	Resistance	20.08	26.08	02.09	09.09	16.09	23.09
Critical	s_*Acinetobacter baumannii*	Carbapenem	0	0	0	0	0	0
	o_Enterobacterales	Cephalosporin	3	5	4	0	2	1
	o_Enterobacterales	Carbapenem	3	3	4	0	1	1
	s_*Mycobacterium tuberculosis*	Rifamycin (rifampin)	0	0	0	0	0	0
High	g_*Salmonella*	Fluoroquinolone	0 (+2)	0 (+3)	0 (+3)	0	0	0
	g_*Shigella*	Fluoroquinolone	1 (+2)	1 (+2)	1 (+2)	0	0	0
	s_*Enterococcus faecium*	Glycopeptide (vancomycin)	0	0	0	0	0	0
	s_*Pseudomonas aeruginosa*	Carbapenem	0	0	0	0	0	0 (+1)
	s_*Neisseria gonorrhoeae*	Fluoroquinolone	0	0	0	0	0	0
	s_*Neisseria gonorrhoeae*	Cephalosporin	0	0	0	0	0	0
	s_*Staphylococcus aureus*	Penicillin beta-lactam (methicillin)	0	0	0	0	0	0
Medium	s_*Streptococcus pyogenes*	Macrolide	0	0	0	0	0	0
	s_*Streptococcus pneumoniae*	Macrolide	0	0	0	0	0	0
	s_*Haemophilus influenzae*	Penicillin beta-lactam (ampicillin)	0	0	0	0	0	0
	s_*Streptococcus agalactiae*	Penicillin beta-lactam (penecillin)	0	0	0	0	0	0
Total number MAGs matching WHO bacterial priority list			4	6	4	1	2	3

Some MAGs are listed more than once, as some organisms are listed multiple times in the WHO priority list for bacteria with different resistance genes.

### 3.5 Added value of shotgun metagenomics: additional pathogens observed

**Figure 4:**
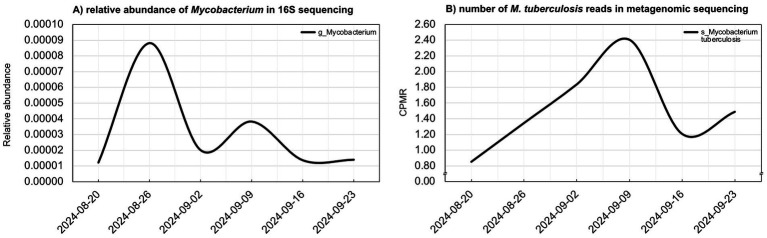
Relative abundance of the genus *Mycobacterium* over time based on **(A)** targeted 16S rDNA analysis and **(B)** shotgun metagenomics. While the abundances are low, the genus can be detected in all samples indicating the constant presence of a source of *Mycobacteria*. The shotgun metagenomic data confirm the presence of *Mycobacterium tuberculosis*.

*Mycobacterium tuberculosis*: The 16S rDNA data revealed the presence of *Mycobacteria* in all samples (Figure 4A). Analyzing the shotgun metagenomes with Kaiju (26) at the level of unassembled reads revealed the presence of a significant number of sequence reads for the obligate pathogen *M. tuberculosis* in all samples (Figure 4B).

**Figure 5:**
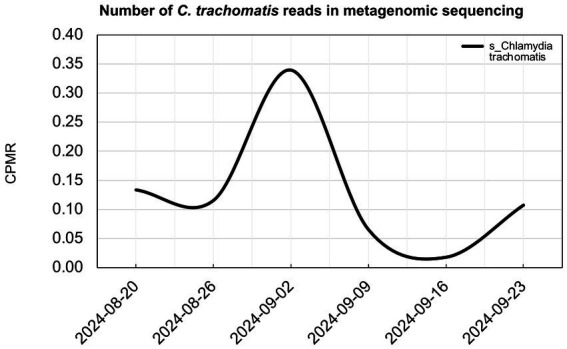
Abundance of *Chlamydia trachomatis* in normalized shotgun metagenomic reads. This bacterium was observed at all timepoints albeit with a low number of reads.

*Chlamydia trachomatis* was also detected in all samples (Figure 5). While the abundance is very low, as *C. trachomatis* is an obligate intracellular pathogen with humans as only host, bacteria detected in wastewater must have originated from an infected person. *C. trachomatis* is a reportable organism. However, there were no reports of *Chlamydia* infections for the examined period.

## 4 Discussion

In this study, we applied multiple molecular approaches to monitor viral and bacterial pathogens, as well as antibiotic resistance genes, in wastewater collected at the influent of a treatment plant in Monheim (serving the adjacent city of Langenfeld) in the district of Mettmann, Germany. The results were compared with surveillance data on notifiable diseases from the corresponding catchment area at the time of sampling. In addition, the implementation and public health actionability of WBE at the regional level were evaluated in collaboration with the Mettmann County Health Department.

### 4.1 Viral pathogens: example SARS-CoV-2

The detection of Viruses via RT-dPCR has the lowest cost and complexity and requires no computational support. For SARS-CoV-2 (Figure 1A) RT-dPCR confirmed the presence of the virus in the population and added information for quantification. As per our expectation, no correlation was observed between cases reported via the traditional surveillance mechanisms and observed viral concentrations in wastewater. This is likely due to reporting requirements for SARS-CoV-2 at the time being reduced, only patients seen by health care providers were reported, as well as widespread immunity among the population resulting dramatically reduced severity of symptoms as well as asymptomatic infections. However, the results highlight the utility of WBE to assess viral load in the local population specially during periods of decreased clinical reporting. The frequency of SARS-CoV-2 lineages reflects national trends during the sampling period (Figure 1B). The high proportion of BA.2.86 at the third time point does not reflect the dominance of the KP.3 variant at that time. This outlier could indicate regional differences or measurement inaccuracies.

Using tiled amplicon sequencing, which entails higher cost and complexity, we confirmed that local viral variants closely reflect national averages. When viral variant patterns are stable, the added value of local genotyping for routine surveillance in our setting is limited. This may differ at major points of entry or in large metropolitan areas, where the introduction of novel, potentially immune-evasive variants could prompt adjustments in healthcare measures, such as updated vaccines or antiviral therapies. In our context, information on SARS-CoV-2 quantity and subtypes is unlikely to have immediate public health impact and does not justify the routine use of tiled amplicon sequencing. Based on our limited data, the emergence of immune-evasive variants is adequately anticipated by national trends. However, SARS-CoV-2 surveillance has been optimized at considerable cost, and this balance may differ for other pathogens, where costs and benefits of local sequencing may vary.

### 4.2 Bacterial pathogens and antibiotic resistance genes

Our results demonstrate that both qPCR and metagenomics can reliably identify ARGs and bacterial pathogens in wastewater (Figures 2–5). The different methods showed largely consistent results while highlighting important differences in sensitivity and resolution.

For bacterial pathogens, the relatively low costs of targeted, 16S rDNA-based metagenomics, combined with the availability of ready-to-use bioinformatics pipelines (19) may support its adoption in certain public health contexts. Using this approach, bacteria resolution remains at the genus level, as an inherit limitation of the method (30–32). At higher costs, reconstructing near-complete genomes from shotgun metagenomic data allows identifying species and even specific strains, as well as capture their antimicrobial resistance determinants. This can facilitate the identification of infection clusters within a population. With this method, we could obtain MAGs for MDRO Enterobacterales early on in our wastewater sampling campaign, and consequently for nearly the entire sampling period (Table 2; Supplementary Table S4). Notably, the presence of such bacteria in wastewater was identified before it was reported through patient-based surveillance. Unfortunately, due to the absence of genotype information from isolates, we cannot directly compare the genotypes observed in case-based surveillance with those recovered via WBE.

Both sequencing strategies additionally detected signals from organisms associated to other reportable diseases, which were not captured by case-based surveillance, such as *Mycobacterium tuberculosis* and *Chlamydia trachomatis*, the latter only identified by shotgun metagenomics.

*C. trachomatis* serotypes L1-L3 became notifiable in Germany in 2023. Finding evidence of this pathogen in wastewater suggests that some infections might have been undiagnosed. Wastewater surveillance could offer a non-invasive way to estimate the regional prevalence of sexually transmitted infections (STIs). Such undetected cases are common and, if untreated, the spread of STIs can cause long-term fertility problems. Such risks could be mitigated by WBE-informed localized screening initiatives. While resource constraints did not allow our study to extend to neighborhood-level source tracking, enabling public health authorities to localize signals to neighborhoods could, in the future, support targeted testing and proactive containment.

Despite the general consistency of detection at the genus level for target and untargeted metagenomics, we noticed some discrepancies. Examples are the absence of the genera *Staphylococcus* and *Chlamydia* in the 16S rDNA analysis, despite the detection of *Staphylococcus aureus* and *Chlamydia trachomatis* by shotgun metagenomics. This may reflect methodological differences between the two approaches: in addition to distinct filtering thresholds and taxonomic classification tools, 16S rDNA sequencing typically relies on substantially lower read counts per sample (100,000 sequences), limiting sensitivity. In contrast, for shotgun metagenomics we acquired approximately 200 million sequences per sample, thus increasing the likelihood of detecting low-abundance taxa.

Considering ARGs exclusively, the results of qPCR and shotgun metagenomics mostly corroborated each other: for example, neither approach detected NDM or IMP, indicating these genes are likely absent in our samples. Quantitative trends were also consistent: *sul1* and *tetO* were among the most abundant genes in metagenomic data and showed correspondingly low CT values in qPCR. On the other hand, VIM was detected by qPCR with a high CT value (~32) but was not captured by shotgun metagenomics, highlighting differences in sensitivity between the methods. Together, these findings indicate that qPCR and shotgun metagenomics provide complementary and reliable approaches for both detection and relative quantification of ARGs in wastewater, with qPCR offering high sensitivity for low-abundance genes and metagenomics enabling broader profiling as an untargeted approach.

It should be noted that NDM, IMP, VIM, and OXA-48-like represent gene families encoding different *β*-lactamase groups with overlapping substrate profiles (Table 1). Enzymes from all four groups confer resistance to penicillins and carbapenems, whereas the metallo-β-lactamases NDM, IMP, and VIM additionally confer resistance to extended-spectrum cephalosporins. Since carbapenem resistance is a hallmark of MDRO outbreaks in hospitals, the fact that these targets were either not detected or detected at very low levels by qPCR and/or metagenomics, suggests these clinically relevant organisms are rare. At the same time, detection aligns with regional case reports indicating at least one patient with carbapenem-resistant Enterobacterales during the study period. These findings illustrate not only the detectability of critical resistance genes but also the capacity to observe differences in abundance, underscoring the potential of wastewater-based epidemiology for early outbreak detection.

In summary, using qPCR, we can reliably, rapidly and with low cost and complexity quantify the abundance of resistance genes in wastewater. However, careful quality control is essential, as small technical variations can affect accuracy and reliability, which is critical for public health interpretation. Additionally, it is not possible to link the resistance information to taxonomic information thus minimizing the information for public health. While the cost and complexity of shotgun metagenomics is significantly higher, it allows linking resistance to species. Future enhancements for sequencing technology will further improve this capability by allowing an even better inclusion of plasmids as one of the main drivers of resistance spread (33, 34). Targeted 16S sequencing occupies an intermediate position, providing scalable microbial community-level insights with moderate cost and complexity. With the cost for sequencing trending downwards, the choice of technology while currently data generation cost-dominated, likely will become analysis cost dominated in the near future.

In our opinion, due to the significant number of resistance genes observed in environmental organisms with no clinical relevance, merely enumerating ARGs does not necessarily add information for health care providers or public health authorities. In this context, qPCR remains valuable for quantifying ARGs with the potential for horizontal transfer to pathogens of clinical or public health significance.

## 5 Conclusions and public health implications

This study contributes to the growing evidence demonstrating that wastewater-based epidemiology can function as a practical extension of routine public health surveillance. By focusing on its applicability in a small district setting, this study moves beyond a proof-of-concept by linking methodological choices to actionable outputs within a real-world surveillance context.

By combining qPCR and metagenomic approaches, we detected clinically relevant pathogens and antimicrobial resistance signals at the population level, including instances not captured by case-based reporting. Signals of carbapenem-resistant Enterobacterales, as well as obligate pathogens such as *Klebsiella pneumoniae*, *Mycobacterium tuberculosis*, and *Chlamydia trachomatis*, indicate silent or under-ascertained transmission which in some cases preceded routine detection.

From a public health perspective, this provides early, population-level intelligence that can guide timely and proportionate responses. For example, the repeated detection of *C. trachomatis* DNA, even in the absence of confirmed cases, underscores the need to renew vaccination for at-risk groups and equip sexual health services with neutral, non-stigmatizing information. Promotion of accessible testing options, including self-sampling and youth-friendly services would be also relevant. Communication should stress that wastewater signals are aggregated and do not indicate individual signals. Similarly, the detection metagenomic reads assigned to *M. tuberculosis* underscore the need for increased clinical vigilance. Tuberculosis (TB) is often identified only at advanced stages in patient-based surveillance, suggesting WBE could support passive TB monitoring in high-risk populations (35). In these two cases, wastewater surveillance is particularly valuable in areas with limited diagnostic access or underreporting.

Early detection of antibiotic-resistant *Klebsiella pneumoniae* provides actionable feedback for hospital stewardship and infection-prevention teams, reinforcing multifactorial interventions such as targeted admission screening for high-risk patients and rigorous environmental hygiene measures.

A central contribution of this work is the systematic comparison of methodological approaches in terms of sensitivity, resolution, cost and public health utility. Based on this, preparedness can be enhanced through a tiered test approach suited to decision-making needs. During routine periods, weekly qPCR of a minimal sentinel panel provides affordable, broad coverage and robust trend monitoring. Periodic targeted 16S rDNA analysis can then screen for broader bacterial shifts, prompting review when genus-level signals change. In response to suspicious trends or suspected outbreaks, shotgun metagenomics offers high-resolution species-level characterization of microbial populations and supports source tracking, particularly when integrated with local or regional genome reference data. Because pathogen abundances in composite samples can be influenced by the sewage system and human behavior, future campaigns should incorporate contextual metadata such as temperature and holiday mobility to stabilize interpretation under atypical conditions. While the wastewater system studied here does not include rainwater, systems with combined sewer networks should account for rainfall effects in data interpretation. Incorporating these contextual variables into machine learning models can help distinguish normal variation from unusual peaks, thereby enhancing early detection of potential outbreak (8, 36).

This study represents the first fully operational integration of WBE into a practical public health workflow in Germany. Unlike previous proof-of-concept studies, our work directly engages public health authorities, ensuring findings are immediately actionable. By bridging research and applied practice, this study proposes a model for effectively using WBE in real-world disease surveillance and policy decision-making.

## Data Availability

The datasets presented in this study can be found in online repositories. The names of the repository/repositories and accession number(s) can be found at: https://www.ebi.ac.uk/ena, PRJEB101125.
