# Supplementary material for: Wastewater-based epidemiology for public health – benefits and trade-offs of different molecular methods for the generation of actionable data in a small-town context
Source: Front Public Health. 2026 Jun 22;14:1828355. doi: 10.3389/fpubh.2026.1828355 (PMC13333637; doi:10.3389/fpubh.2026.1828355)
Supplement: Supplementary file 1 [file Supplementary_file_1.docx]

Supplementary Information

This study was performed in the context of the WBEready project (https://wbeready.de/). WBEready is a research initiative funded by the German Federal Ministry of Health, which aims at advancing wastewater-based epidemiology (WBE) as a routine component of public health surveillance. Building on the national program AMELAG [“*Abwassermonitoring für die epidemiologische Lagebewertung*”, in English: “Wastewater Monitoring for Epidemiological Situation Assessment”(1)], the project seeks to expand Germany’s wastewater surveillance beyond SARS-CoV-2, to include a broader range of viral pathogens and antimicrobial resistance markers. Its objective is to develop a scalable and adaptive framework for WBE that supports preparedness and decision-making in public health systems.

To achieve this, WBEready addresses key analytical, epidemiological and operational questions, including the suitability of new targets for wastewater monitoring, the interpretation of WBE data for surveillance purposes and its integration into institutional workflows.

Within the WBEready project, wastewater surveillance methods were developed and evaluated through a combination of 24 h composite sampling at treatment plant influents and targeted sub-catchment sampling within sewer networks, followed by molecular detection using qPCR-based assays and sequencing-based approaches (targeted and metagenomic) for the identification of viral pathogens, bacteria and antimicrobial resistance genes. Method performance is assessed through inter-laboratory comparisons and real-world validation, and complemented by computational approaches to support data interpretation and integration into public health decision-making.

## Supplementary Table 3. Detailed read counts and counts per million reads (CPMRs) from metagenomic sequencing for the antibiotic resistance genes described in this study.

## Supplementary Table 4. Species-level information for metagenome-assembled genomes (MAGs) related to WHO priority pathogens.

**References**

1. Marquar N, Puetz P, Buchholz U, Exner T, Fretschner T, Greiner T, Helmrich M, Lukas M, Marty M, Obermaier N, Saravia Arzabe C, Schattschneider A, Schneider B, Selinka H-C, Ullrich A, Walther B, Braun U, Schumacher J: SARS-CoV-2-Abwassersurveillance in Deutschland im Rahmen des Projekts AMELAG Epid Bull 2024;34:16-26. DOI 10.25646/12208
