# Supplementary material for: Wastewater-based epidemiology for public health – benefits and trade-offs of different molecular methods for the generation of actionable data in a small-town context
Source: Front Public Health. 2026 Jun 22;14:1828355. doi: 10.3389/fpubh.2026.1828355 (PMC13333637; doi:10.3389/fpubh.2026.1828355)
Supplement: Supplementary file 2 [file Table_1.docx]

**Supplementary Table 1. qPCR primers for antibiotic resistance**

| **Target** | **Primer 1** | **Sequence Primer 1** | **Primer 2** | **Sequence Primer 2** | **Source** |
| --- | --- | --- | --- | --- | --- |
| *tetA* | tetA_1 | GCTACATCCTGCTTGCCTTC | tetA_2 | CATAGATCGCCGTGAAGAGG | (1) |
| *sul1* | sul1_1 | GCTATTGGTCTCGGTGTCGC | sul1_2 | GCATGATCTAACCCTCGGTCT | (2) |
| *bla*VIM-1 | blaVim-1_1 | GTACGCATCACCGTCGACAC | blaVim-1_2 | TGACGGGACGTATACAACCAGA | (3) |
| *ermB* | ermB_1 | GGTTGCTCTTGCACACTCAAG | ermB_2 | CAGTTGACGATATTCTCGATTG | (4) |
| *bla*OXA-48 | blaOXA-48_1 | TGTTTTTGGTGGCATCGAT | blaOXA-48_2 | GTAAAAATGCTTGGTTCGC | (5) |
| *tetB* | tetB_1 | ACACTCAGTATTCCAAGCCTTTG | tetB_2 | GATAGACATCACTCCCTGTAATGC | (6) |
| *blaNDM-1* | blaNDM-1_1 | GGCGGAAGGCTCATCACGA | blaNDM-1_2 | CGCAACACAGCCTGACTTTC | (7) |
| *tetO* | tetO_1 | GATGGCATACAGGCACAGACC | tetO_2 | GCCCAACCTTTTGCTTCACTA | (8) |
| *bla*IMP | blaIMP_1 | AAGTTAGTCAATTGGTTTGTGGAGC | blaIMP_2 | CAAACCACTACGTTATCTGGAGTGTG | (3) |

**References**

1. Zhang S, Gu J, Wang C, Wang P, Jiao S, He Z, Han B. Characterization of antibiotics and antibiotic resistance genes on an ecological farm system. J Chem. 2015.
2. Zhao J, Xu Y, Liu W, Ni W, Wei C, Wang R, Liu Y, Liu Y. Surveillance of dihydropteroate synthase genes in *Stenotrophomonas maltophilia* by LAMP: implications for infection control and initial therapy. Front Microbiol. 2016;7.
3. Bisiklis A, Papageorgiou F, Frantzidou F, Alexiou-Daniel S. Specific detection of *blaVIM* and *blaIMP* metallo-β-lactamase genes in a single real-time PCR. Clin Microbiol Infect. 2007;13.
4. Mackie RI, Koike S, Krapac I, Chee-Sanford JC, Maxwell S, Aminov RI. Molecular ecology of macrolide-lincosamide-streptogramin B methylases in waste lagoons and subsurface waters associated with swine production. Environ Microbiol. 2009.
5. Monteiro J, Widen RH, Pignatari ACC, Kubasek C, Silbert S. Rapid detection of carbapenemase genes by multiplex real-time PCR. J Antimicrob Chemother. 2012;67:906–909.
6. Peak N, Knapp CW, Yang RK, Hanfelt MM, Smith MS, Aga DS, Graham DW. Abundance of six tetracycline resistance genes in wastewater lagoons at cattle feedlots with different antibiotic use strategies. Environ Microbiol. 2007;9:143-151.
7. Xiang T, Chen C, Wen J, Liu Y, Zhang Q, Cheng N, Wu X, Zhang W. Resistance of *Klebsiella pneumoniae* strains carrying *blaNDM-1* gene and the genetic environment of *blaNDM-1*. Front Microbiol. 2020;11.
8. Luo Y, Mao D, Rysz M, Zhou Q, Zhang H, Xu L, Alvarez PJJ. Trends in antibiotic resistance genes occurrence in the Haihe River, China. Environ Sci Technol. 2010;44.
