# Supplementary material for: Wastewater-based epidemiology for public health – benefits and trade-offs of different molecular methods for the generation of actionable data in a small-town context
Source: Front Public Health. 2026 Jun 22;14:1828355. doi: 10.3389/fpubh.2026.1828355 (PMC13333637; doi:10.3389/fpubh.2026.1828355)
Supplement: Supplementary file 3 [file Table_2.docx]

Supplementary Table 2. Results of digital RT-PCR and qPCR analyses performed in this work

a-1. dPCR – SARSCoV-2 (copies/µl RNA)

| Sample date | SARS-CoV-2 N1 | SARS-CoV-2 N2 |
| --- | --- | --- |
| 20.08.2024 | 0.00 | 0.00 |
| 26.08.2024 | 37.86 | 55.84 |
| 02.09.2024 | 22.49 | 34.65 |
| 09.09.2024 | 27.59 | 50.32 |
| 16.09.2024 | 30.91 | 49.80 |
| 23.09.2024 | 44.36 | 68.76 |

a-2. dPCR – SARSCoV-2 (copies/L wastewater)

| Sample date | SARS-CoV-2 N1 |
| --- | --- |
| 20.08.2024 | 0 |
| 26.08.2024 | 6.6x10^5^ |
| 02.09.2024 | 3.9x10^5^ |
| 09.09.2024 | 4.8x10^5^ |
| 16.09.2024 | 5.4x10^5^ |
| 23.09.2024 | 7.8x10^5^ |

b. qPCR – Antimicrobial resistance genes (CT values)

| Sample date | VIM | OXA-48-like | *sul*1 | ErmB | *tet*A | *tet*B | *tet*O |
| --- | --- | --- | --- | --- | --- | --- | --- |
| 20.08.24 | 31.55 | 33.21 | 14.58 | 13.26 | 17.08 | 20.4 | 13.53 |
| 26.08.24 | 32.05 | 29.11 | 13.82 | 14.75 | 16.5 | 20.5 | 15.33 |
| 02.09.24 | 29.47 | 30.57 | 14.97 | 14.22 | 17.28 | 19.9 | 14.57 |
| 09.09.24 | 31.86 | 30.68 | 15.26 | 18.01 | 17.91 | 23.35 | 20.13 |
| 16.09.24 | 31.3 | 31.06 | 14.26 | 15.64 | 17.05 | 20.74 | 16.5 |
| 23.09.24 | 30.94 | 29.67 | 15.35 | 16.57 | 18.62 | 22.06 | 17.41 |
